# Binasal Field Defect in Non-arteritic Anterior Ischemic Optic Neuropathy

**DOI:** 10.7759/cureus.43722

**Published:** 2023-08-18

**Authors:** Syarifah Nur Humaira Syed Mohd Khomsah, Julieana Muhammed, Wan-Hazabbah Wan Hitam, Juhara Haron

**Affiliations:** 1 Department of Ophthalmology and Visual Science, School of Medical Sciences, Universiti Sains Malaysia, Kota Bharu, MYS; 2 Ophthalmology Clinic, Hospital Universiti Sains Malaysia, Kota Bharu, MYS; 3 Department of Radiology, School of Medical Sciences, Universiti Sains Malaysia, Kota Bharu, MYS

**Keywords:** binasal hemianopia, bilateral optic disc atrophy, optic disc swelling, non-arteritic anterior ischemic optic neuropathy, binasal field defect

## Abstract

Non-arteritic anterior ischemic optic neuropathy (NAION) is the most common cause of optic neuropathy in older adults and is usually associated with an altitudinal visual field defect. Binasal hemianopia is a rare visual field presentation, and most causes are due to ocular pathology instead of brain pathology. It is an infrequent finding in NAION. We report a rare presentation of binasal hemianopia visual field defect in a patient with NAION. This a case of an elderly lady with underlying uncontrolled type 2 diabetes mellitus, hypertension, and dyslipidemia who presented with a sudden onset of painless blurring of vision in the left eye. She had a similar episode of blurred vision involving the other eye two years ago. Her visual acuity was reduced in both eyes. Humphrey visual field showed a binasal field defect. Fundoscopy showed mild hyperemic optic disc swelling in the left eye and a pale disc in the right eye. The CT scan and MRI were normal. She was co-managed with the medical team to control her systemic risk factors. Although NAION is the most common cause of optic neuropathy in older adults, binasal hemianopia is a rare visual field presentation in NAION. The history and assessment from this case add important information toward diagnosing NAION.

## Introduction

Ischemic optic neuropathy is the most common optic nerve disorder in patients over the age of 50. Ischemic optic neuropathy is generally categorized as anterior or posterior and as arteritic or non-arteritic. Anterior involvement is common with both arteritic and non-arteritic ischemic optic neuropathy (NAION). NAION is the most common form of ischemic optic neuropathy [1]. It is an idiopathic, ischemic insult of the optic nerve head characterized by acute, monocular, painless visual loss with optic disc swelling. Visual field defects in NAION may follow any pattern related to optic nerve damage, but altitudinal loss, usually inferior, occurs in the majority, ranging from 55% to 80%, of reported cases [2,3]. The risk of bilateral NAION ranges from 15% to 25% according to several studies [4]. This is a report of a rare case of binasal field defect secondary to NAION.

## Case presentation

A 58-year-old lady with underlying type 2 diabetes mellitus, hypertension, and dyslipidemia presented with sudden onset blurring of vision in the left eye for two weeks. It was painless and gradually worsening. On presentation, she reported central scotoma. The color vision was normal, and she had no history of metamorphopsia. She did not report any headache, nausea, or vomiting. There was also no history of trauma or neurological symptoms. She had a similar episode of blurred vision involving the right eye two years ago. At the point of assessment, she had a right nasal field defect and was diagnosed with right NAION. There was no specific treatment given except for close monitoring of her diabetes, hypertension, and hyperlipidemia. Thus, she was advised to come for regular follow-ups.

**Figure 1:**
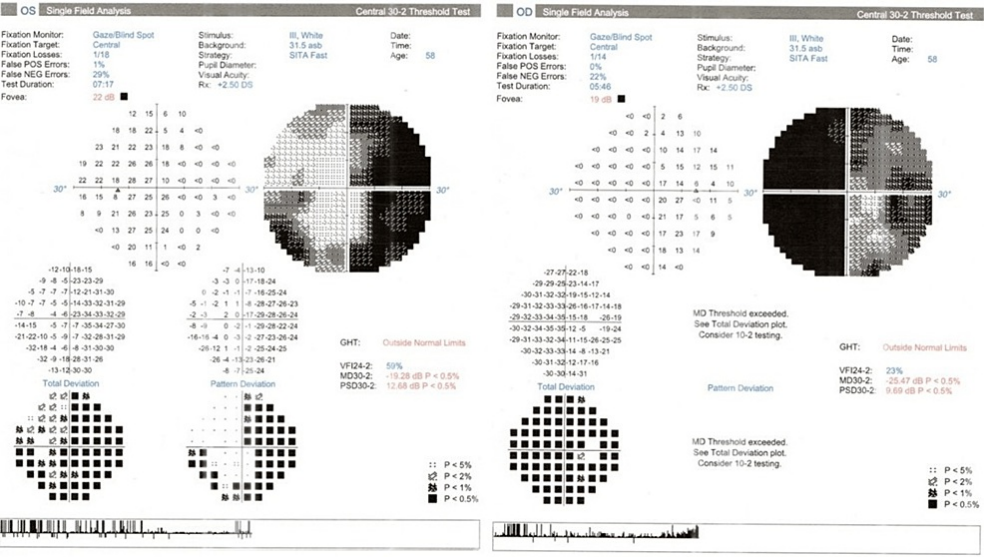
Humphrey visual field showing binasal field defect.

**Figure 2:**
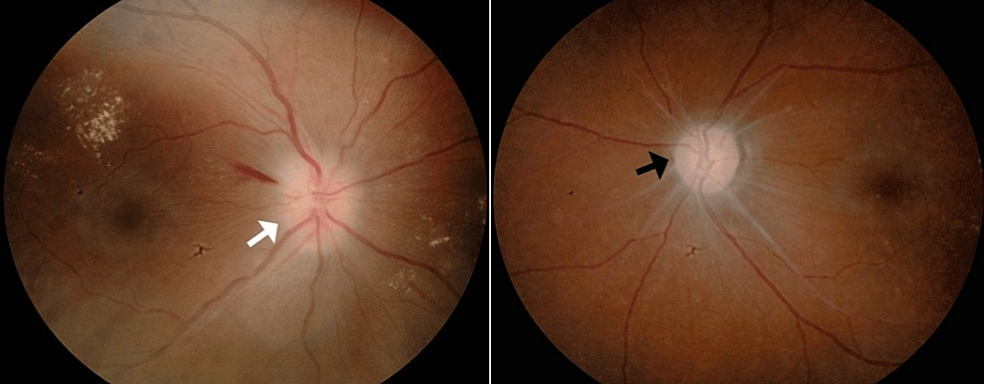
Right hyperemic optic disc swelling (white arrow). Left pale optic disc with sclerosed vessels surrounding the disc (black arrow).

During follow-up, her visual acuity was 6/60 in both eyes. Humphrey visual field showed binasal field defect (Figure 1). The color vision and light brightness were reduced bilaterally. The pupillary reaction was normal bilaterally. Both anterior segments were unremarkable. The intraocular pressure was within the normal range (14 mmHg) in both eyes. Fundoscopy showed right hyperemic optic disc swelling with flame-shaped hemorrhages at the superior-temporal region. There were also multiple scattered exudates temporal to the macula. The left disc was pale, with sclerosed vessels surrounding it (Figure 2).

**Figure 3:**
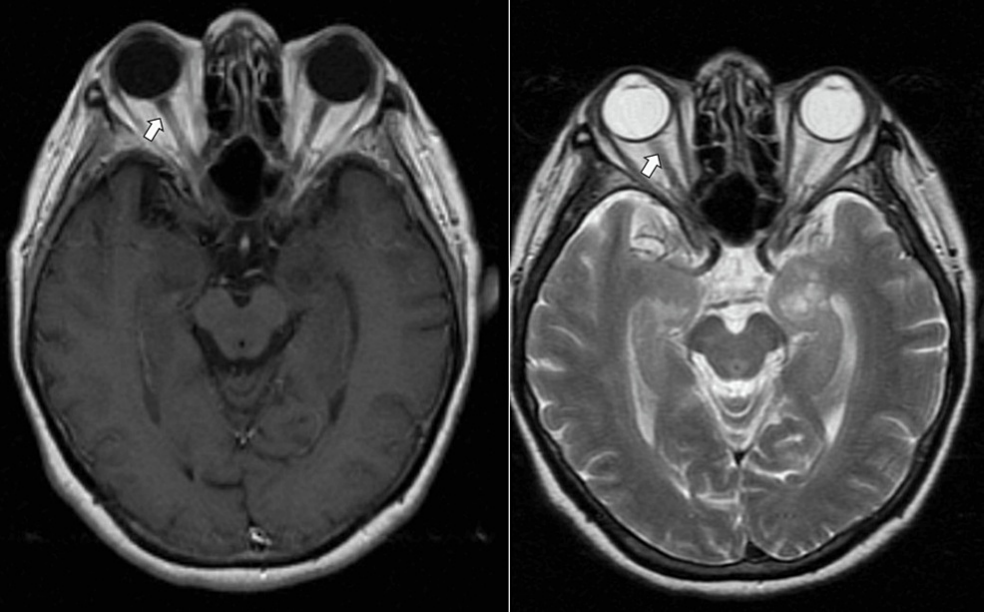
MRI showing bilateral normal optic nerve (white arrow).

Her systemic examination findings were unremarkable. Her blood pressure was 170/82 mmHg. There were no signs of neurological deficits or cranial nerve palsies. Her cardiovascular and respiratory system examination revealed normal findings. There was no mass palpable per abdomen and breast. The hemoglobin level and total white cell count were within normal limits. The renal and liver functions were also normal. The erythrocyte sedimentation rate and C-reactive protein were not elevated with a Mantoux test of 0 mm. Other serologic infective screening for toxoplasmosis, cytomegalovirus, and syphilis were non-reactive. The chest X-ray revealed normal findings. She was found to have uncontrolled diabetes mellitus, as evidenced by her high serum hemoglobin A1c (HbA1c) of 9.7%. Her fasting lipid profile was elevated. A CT scan of the brain and orbit revealed normal findings. MRI of the brain and orbit showed both optic nerves were symmetrical with no abnormal enhancement (Figure 3).

**Figure 4:**
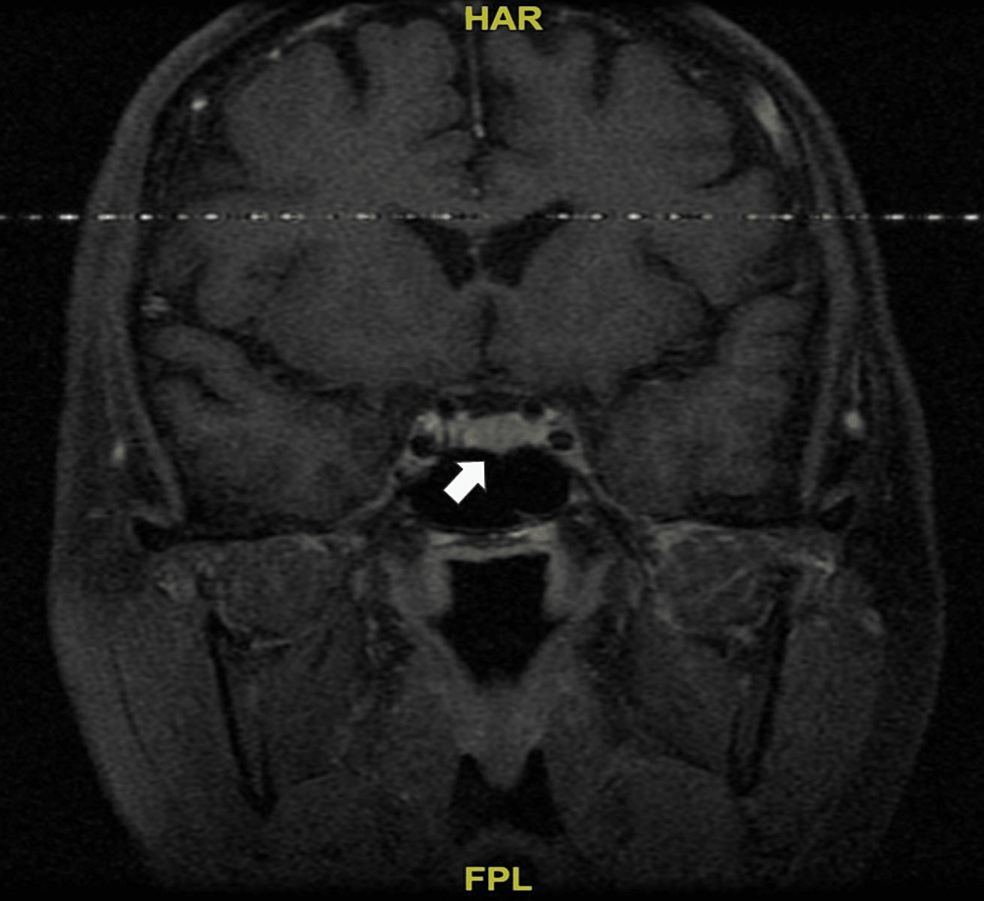
Normal chiasm (white arrow).

Optic chiasm was normal in appearance (Figure 4).

**Figure 5:**
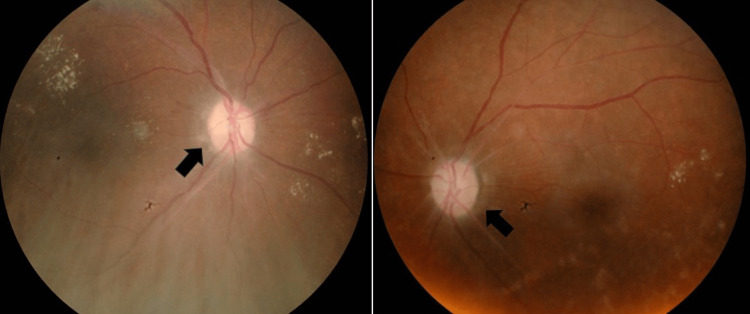
Bilateral optic atrophy on follow-up (black arrow).

As a result, she was diagnosed with bilateral NAION secondary to her uncontrolled diabetes mellitus, hypertension, and hyperlipidemia. Upon diagnosis, oral mecobalamin 500 mg OD was started and she was referred to the medical team for further optimization of her medication. She was subjected to regular follow-ups at the eye clinic. During subsequent follow-ups, her visual acuity remained 6/60 in both eyes with a binasal visual field defect. Both fundi showed a marked pale disc in both eyes (Figure 5).

The patient was informed about her visual prognosis and subjected to a low-vision rehabilitation program.

## Discussion

Patients with NAION typically present with monocular vision loss that develops over hours to days, often manifesting upon awakening in up to 73% of cases. Some individuals may experience altitudinal vision loss or a scotoma, and the visual impairment tends to progress initially and stabilizes within one to two weeks. It is unusual for both eyes to be affected simultaneously, with prior occurrences preceding the current episode being common. However, in our patient’s case, bilateral visual complaints and binasal hemianopia (BNH) were observed, and further investigations were done to rule out other potential causes such as infection, infiltration, or compression-related optic disc swelling.

BNH is a rare occurrence that is typically associated with ocular pathologies rather than brain pathologies. In a study by Selena et al., binasal visual field defects were observed in eight out of 100 patients who were referred for neuro-ophthalmologic examination. Contrary to the initial suspicion of intracranial causes, the study revealed that two patients had ischemic optic neuropathy as the cause of BNH, while the remaining cases were attributed to optic nerve drusen, glaucoma, congenital optic nerve pits, and retinitis pigmentosa. Hence, when encountering patients with binasal field defects, it is crucial to consider ocular causes as the primary etiology rather than intracranial factors [5].

BNH can be caused by ocular lesions (e.g., keratoconus, glaucoma, drusen, retinitis pigmentosa), optic nerve lesions (e.g., syphilitic optic neuropathy, ischemic optic neuropathy, internal carotid artery aneurysm, atherosclerosis, pituitary apoplexy, pneumosinus dilatans, olfactory groove meningioma), and intracranial lesions (e.g., brain tumor, elevated intracranial pressure, occipital lobe lesions). Tawara et al. reported bilateral BNH caused by bilateral optic perineuritis secondary to sarcoidosis. The authors suggested that visual loss in these patients was due to secondary ischemic optic neuropathy caused by compression from the thickened optic nerve sheath [6].

NAION commonly presents with an inferior altitudinal visual field defect, but its prevalence varies widely in reported cases (25-79%). In a study of 312 eyes, only 8% showed an absolute inferior altitudinal defect, while the most common defect observed was an inferior nasal defect (22%) [7]. Other reported defects included central, centrocecal, and arcuate scotomas, as well as generalized depression. BNH presentation in our patient was considered atypical.

Binasal visual field defects can have different causes. Glaucoma is the most common cause of general binasal defects [5,8-10], while neurologic binasal hemianopias are typically related to optic chiasm issues such as pituitary adenoma, ischemia, or aneurysm [11-13]. Neurologic BNH defects are quite rare. Studies found that in cases of intracranial tumors, only 5-6% exhibited nasal hemianopia [5], and in post-stroke patients with visual field loss, only 0.2% had BNH [14]. BNH appears to occur equally in men and women at an average age of approximately 44 years [5]. The occurrence of BNH other than in this range suggests the rare and varied nature of the underlying causes of BNH [5,6,13-17].

BNH exhibits varying visual characteristics, with normal or reduced visual acuity, pupil response, and color vision depending on the underlying cause. It is crucial to rule out ocular causes, particularly glaucoma, which is the most common culprit, before resorting to neuroimaging or lab tests [5,10,12].

Regrettably, there is currently no proven therapy to alleviate visual impairment in NAION [18]. Patients with high blood pressure should have their blood pressure gradually lowered to avoid precipitating NAION [19]. Despite extensive research, there is no scientifically proven evidence of neuroprotective agents benefiting NAION in human clinical studies [19]. Additionally, there is no effective therapy for preventing NAION in the fellow eye [18]. While controlling underlying vasculopathy risk factors is vital for cardiovascular health, its impact on reducing NAION recurrence remains uncertain. A significant proportion of NAION patients experience visual impairment, making them potential candidates for visual rehabilitation services and the assistance of low-vision aids.

## Conclusions

NAION is the leading cause of optic neuropathy in older adults characterized by sudden, painless, monocular vision loss, commonly upon waking from sleep. Clinical signs include optic disc swelling, decreased visual acuity, an afferent pupillary defect, and an inferior altitudinal visual field defect. BNH, a rare visual field presentation, is predominantly associated with ocular rather than brain pathology. Additional evaluation is recommended for patients with atypical features to explore alternative causes.
